# Misclassification of RhD variants among pregnant women: a systematic review

**DOI:** 10.25122/jml-2023-0004

**Published:** 2023-07

**Authors:** Amani Yousef Owaidah, Lamya Zohair Yamani

**Affiliations:** 1 Department of Clinical Laboratory Sciences, College of Applied Medical Sciences, Imam Abdulrahman bin Faisal University, Dammam, Saudi Arabia

**Keywords:** RhD variants, anti-D alloimmunization, HDFN, pregnant women

## Abstract

The D antigen of the Rh blood group is considered clinically significant due to its ability to cause hemolytic transfusion reactions and hemolytic disease in the fetus and newborn. This systematic review discusses the prevalence of RhD variants among pregnant women and the importance of including RhD genotyping for prenatal testing to detect RhD variants and prevent anti-D alloimmunization. A comprehensive literature search was conducted using scientific search engines, including PubMed and MEDLINE databases, with the keywords 'anti-D alloimmunization', 'RhD variant', and 'pregnant women.' The review adhered to the PRISMA guidelines. Meta-analysis was performed using MedCalc version 20. A significance level of p≤0.05 was considered statistically significant for all two-tailed tests. The meta-analysis included four articles that met the inclusion criteria. The total prevalence of RhD positivity (RhD+) was 61% (95% CI:34%–85%). The prevalence ranged from 22% to 82%, indicating a high degree of heterogeneity between studies (I2=98.71%, p<0.0001). The overall prevalence of D variants was 15% (95% CI, 9%–23%) with a prevalence of 0.05% to 100%, showing a high degree of heterogeneity between studies (I2=99.89%, p<0.0001). Anti-D alloimmunization could occur in pregnant women with some types of RhD variants. All four studies focused on molecular testing of samples showing inconsistent or weak results with at least two anti-D antibodies using serological methods.

## INTRODUCTION

The RhD antigen, second in clinical significance after the ABO blood group system, is highly immunogenic and known for its potential to cause hemolytic transfusion reactions and hemolytic disease of the fetus and newborn (HDFN), particularly in obstetric patients [1-3]. Approximately 85% of individuals are identified as RhD-positive, and the remaining 20% are RhD-negative [4]. According to most authors, women with typical weak D types do not require RhD immunoprophylaxis due to the low risk of RhD immunization. Women with weak D types 1, 2, and 3 [5-7] and weak D could safely receive D+ RBC units [8]. Conversely, partial D women are classified as D-negative and receive prenatal care and RhD immunoprophylaxis [9]. To ensure that such women undergo immunoprophylaxis in their second trimester and/or postpartum, anti-D reagents are purposely chosen to categorize pregnant DVI carriers as D-. Different populations have different distributions of D variations, and the ability to identify them depends on the D typing reagents used [10].

Among women that are RhD+ or RhD-, some of these individuals fall into a category known as RhD variants. These RhD variants consist of more than 450 variants between weak D, partial D, and DEL phenotypes [11]. These terminologies surrounding RhD variants lead to confusion in RhD result interpretation. In prenatal tests, pregnant women grouped as RhD- are considered eligible to receive RhD immunoglobulin (RhIG) as a preventive measure for HDFN [12, 13]. However, some women who are RhD are mistakenly grouped as RhD+ or weak D and are not eligible for receiving RhIG, which puts them at risk of RhD alloimmunization and their fetuses at risk of HDFN. Although most weak D variants, such as weak D type 1, type 2, and type 3, do not form anti-D, other types, such as DAR and weak D type 4.2, have been reported to form anti-D [13]. In the current systematic review and meta-analysis, we reviewed the literature for reported cases of pregnant women with RhD variants who were initially reported as RhD positive, weak D, or had discrepant results.

## MATERIAL AND METHODS

### Study design

The current study was conducted as a systematic review and meta-analysis following the guidelines outlined in the Preferred Reporting Items for Systematic Reviews and Meta-Analyses (PRISMA) [14].

### Database and search strategy

A comprehensive and systematic search was performed in PubMed and MEDLINE databases to identify relevant published literature. The search terms used included 'anti-D alloimmunization', 'RhD variant', and 'pregnant women.' We included articles published between 2011 and 2022 with no regional limitation. The authors manually carried out the identification, screening, selection, and data extraction of the studies.

### Inclusion and exclusion criteria

The inclusion criteria for the study were as follows: 1) study population involved only pregnant women; 2) pregnant women with discrepancies in the serological results; 3) pregnant women grouped as RhD positive; 4) original research articles. Duplicates were removed, and the abstracts of the remaining articles were screened for inclusion criteria. Studies were excluded if they met any of the following criteria: 1) sample population did not consist of pregnant women; 2) pregnant women had an RhD negative phenotype; 3) articles were classified as reviews, commentaries, editorials, case studies, or letters to editors.

### Selection of articles and data extraction

After applying the eligibility criteria, relevant articles were chosen for full-text screening. Each author independently evaluated the eligibility evaluation and article screening process. An impartial third party decided whether there were discrepancies between the authors. The title and abstract of the article were used as initial screening criteria for the articles. The title and abstract of the case were not included in the secondary screening of the articles because they were not related to the current investigation. The following data were extracted from the selected articles, which include: 1) first author; 2) year of publication; 3) country; 4) type of study; 5) type of RhD variants.

### Statistical analysis

A meta-analysis was conducted to calculate the prevalence of RhD-positive, discrepant serological results, and RhD variants. The exact 95% confidence interval (CI) was estimated in each study. Both fixed-effects and random-effects models were employed. The fixed effect model provides a standard effect size for the identified population (i.e., RhD variants among serologically discrepant results among the number of patients selected). The studies selected varied considerably in region, population size, and other factors. In addition, the fixed effect model gives a descriptive analysis of the included studies but not the inference of a wider population. Thus, a random effect model was also applied. In fixed effect, the true effect size is assumed to be the same in all the studies. However, the true effect size in the random model is assumed to vary between the studies. Hence, the mean of the variance is considered in the random model to avoid the over-influence of the small or larger population. A random effect model was then used to pool the data based on the RhD+ve, discrepant, and D variants.

Under the fixed-effect model, the null hypothesis assumed zero effect of discrepancy in each study. Under the random-effect model, the mean effect of RhD discrepancy was assumed to be zero. Overall statistical heterogeneity was evaluated using I2 statistics. Publication bias was performed using the Egger test considering publication delay, data outcome, methodology description, analysis, true heterogeneity, and artefacts. The results were scored as 1-low risk, 2-medium risk, and 3-high risk. The same t-test was used to calculate significance. All meta-analyses were performed using MedCalc version 20. Differences with a p-value of 0.05 or above were considered statistically significant in all two-tailed tests.

## RESULTS

### Literature search

**Figure 1:**
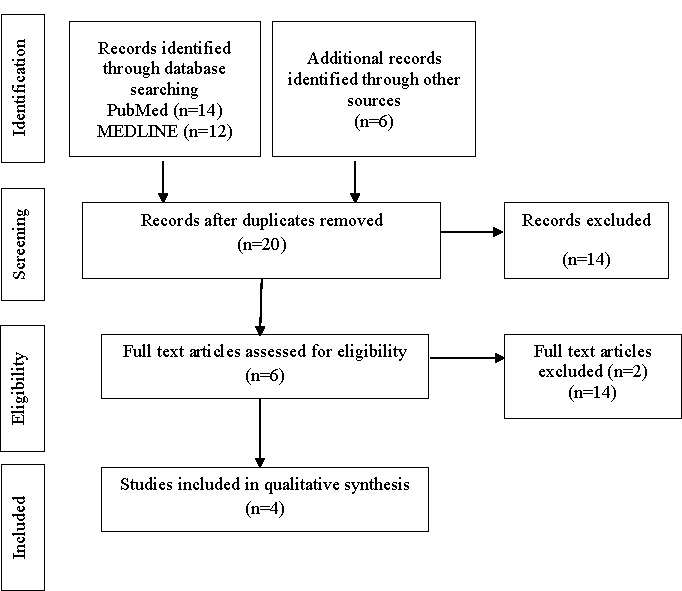
Flowchart showing the process of study selection

A total of 32 articles were initially retrieved using the search strategy. In the first round of selection, 12 duplicate articles were eliminated. From the 20 articles, 14 were excluded in the second round of screening, and 6 remained for full-text review. Following the full-text review, only four articles met the inclusion criteria and were included in the study. Figure 1 summarizes the literature search process.

### Characteristics of the studies included

Four studies met the inclusion criteria [15-18] (Table 1). The selected articles were published between 2016 and 2019. The studies were conducted in Brazil, Canada, Croatia, and France. The four studies selected focused on the molecular testing samples with at least one anti-D antibody using serological methods. Among the four studies, one study by Laget *et al*. evaluated the cost of *RHD* genotyping in pregnant women to reserve RhD- negative blood in transfusion cases and for RhIG prophylaxis [17].

**Table 1:** Characteristics of the studies included

Criteria	Bub *et al*. (2016) [15]	Clarke *et al*. (2016) [16]	Laget *et al*. (2019 [17]	Krstic *et al*. (2017) [18]
Total number of pregnancies tested	21353	608486	NR	12689
Study population	European, African, and Native-American ancestry	British Columbia, Alberta, Saskatchewa, and Manitoba	Rhône-Alpes Auvergne, Pyrenees-Mediterranean, Mediterranean Lampstand Réunion Island.	Region of Croatia
Type of Analysis	hemagglutination using anti-D MoAbs with IgM	hemagglutination with IgM and IgG anti-D MoAbs	NR	hemagglutination with IgM anti-D
	Non-reactive samples confirmed with Anti-D IgG using an indirect antiglobulin test	non-reactive sample with novaclone anti-D	NR	tube testing with 5 MoAbs anti-D; Ortho anti-D, DiaClon anti-D, mono Gnost anti-D, Novaclone anti-Dand MG MonoGnost anti-D
	discrepant serological results confirmed by multiplex PCR for RHD gene hybrid alleles and direct automated sequence of RHD gene	discrepant serological results with genotyping using the BLOODChip platform for the RHD gene and PCR for the RHD exons and flanking regions	NR	PCR and RHD genotyping
Confirmed RhD variants	21249 (99.51%)	608135 (99.94%)	NR	12632 (99.55%)
No of discrepant result	104 (0.49%)	351 (20 showed no variant) and 330 showed the presence of the RHD gene by genotyping assay (0.06%)	273	57 (0.45%)

NR - not reported, MoAbs- monoclonal antibodies

### Identification of RhD variants

RhD variants in the selected studies were initially identified using hemagglutination assay using anti-D monoclonal antibodies with IgM, IgG, or both. Non-reactive samples were confirmed through an indirect antiglobulin test using anti-D IgG. Krstic *et al*. [18] employed tube testing with five monoclonal antibodies of Anti-D such as Ortho anti-D, DiaClon anti-D, MonoGnost anti-D, Novaclone anti-D, and MG MonoGnost anti-D. Discrepant serological results were confirmed by molecular *RHD* genotyping. Bub *et al*. [15] used multiplex PCR for RHD gene hybrid alleles and a direct automated sequence of the RHD gene. Clarke *et al*. [16] performed genotyping using the BLOODChip platform and PCR for RHD exons and flanking regions. The identified RhD variants included weak D, partial D DEL, and other phenotypes. Table 2 summarizes the most common variants identified with percent prevalence.

**Table 2:** Prevalence of RhD variants among discrepant results

Categories	Bub *et al*. (2016) [15]	Clarke *et al*. (2016) [16]	Laget *et al*. (2019) [17]	Krstic *et al*. (2017) [18]
No. of discrepant result	104	330	273	57
Weak D-type 1	9 (8.65%)	119 (36.06%)	85 (31.14%)	24 (42.11%)
Weak D-type-2	12 (11.54%)	45 (13.64%)	61 (22.34%)	Not reported
Weak D-type-3	2 (1.92%)	49 (14.85%)	16 (5.86%)	18 (31.58%)
Weak partial 4	51 (49.04%)	NR	33 (12.09%)	10 (17.54%)
DAR	12 (11.54%)	NR	52 (19.05%)	NR
DVI	10 (9.62%)	NR	NR	NR
Other weak D	8 (7.69%)	117 (35.45%)	26 (9.52%)	53 (92.98%)
Rare alleges detected	Weak D-type 38 (6%)	17 mutations identified	4-DAU4	43-heterozygous type1/3
	Weak D-type 45 (1%)	22 heterozygotes of RHD mutants	5-DAU5	10-type Va
	Weak D-type-67 (1%)	67 RHD variants	1 each-DIIIb, DFR1, type-25, type-29, type-51, type 51	
		11 unresolved variants	2-type 18	

NR - Not reported

The study by Bub *et al*. [15] showed that the weak variant RhD 4 or 4.3 accounted for 49.04% of the discrepant cases, compared to only 12.09% of the cases in Laget *et al*. [17] and 17.54% in Krstic *et al*. [18]. On the other hand, the RhD variant DAR was identified in 19.05% of discrepant cases in Laget *et al*. [17] and 11.54 % in the study by Bub *et al*. [15]. Other variants, such as weak type 1, 2, and 3 D, were identified in all four studies. However, these variants were not reported to induce anti-D production.

### Prevalence of RhD-positive

Table 3A shows the prevalence of RhD+ve in the studies except for Laget *et al*. [17] since the manuscript did not describe the total sample analyzed. The three selected studies show a narrow range of CI, indicating greater precision. Under the fixed effect model, smaller studies [15, 18] are assigned about 3.32 and 1.98% weight, while larger studies [16] are assigned 94.7% weight. The logic of the fixed model provides a good estimate of the effect provided by larger studies compared to less reliable smaller studies. In contrast, the random-effects model shows almost similar weight in each study, indicating the effective size for its unique population. The total prevalence of RhD+ve was 99.71% (95% CI, 99.20%–99.96%). The heterogeneity test revealed a high level of heterogeneity with an I^2^ value of 98.71% (p<0.0001) (Table 3B).

**Table 3A:** Prevalence of RhD+ve

Study	Sample size	Proportion (%)	95% CI	Weight (%)	
				Fixed	Random
**Bub *et al*. (2016)** [15]	21353	99.513	99.41 to 99.60	3.32	33.29
**Clarke *et al*. (2016)** [16]	608486	99.942	99.94 to 99.95	94.7	33.67
**Krstic *et al*. (2017)** [17]	12689	99.551	99.42 to 99.66	1.98	33.03
**Total (fixed effects)**	642528	99.93	99.92 to 99.94	100	100
**Total (random effects)**	642528	99.712	99.20 to 99.97	100	100

**Table 3B:** Test for heterogeneity

Q	261.2294
DF	2
Significance Level	p<0.0001
I^2^ (inconsistency)	99.23%
95% CI for I^2^	98.80 to 99.51

### Prevalence of serological discrepant results

Table 4A shows 4 studies that evaluated the prevalence of discrepant results included in the meta-analysis. All four studies show a narrow range of CI, indicating greater precision. Compared to other studies, the fixed model shows a good estimate of the discrepant result in the case of Clarke *et al*. [16]. In contrast, the random-effects model shows almost similar weight, indicating the effective size for its unique population. The heterogeneity of the studies is shown in Table 4B. Consequently, there was significant heterogeneity between the studies for the discrepant serological results among the RhD variants. The overall prevalence of discrepant was 15.37% (95% CI, 8.8%–23.38%), and the discrepant prevalence varied from 0.05% to 100% to show a high degree of heterogeneity between studies (I^2^=99.89%, p<0.0001) (Table 4B).

**Table 4A:** Prevalence of discrepant results

Study	Sample size	Proportion (%)	95% CI	Weight (%)	
				Fixed	Random
**Bub *et al*. (2016)**[15]	21353	0.487	0.39 to 0.59	3.32	25.5
**Clarke *et al*. (2016)**[16]	608486	0.0542	0.049 to 0.060	94.66	25.53
**Laget *et al*. (2019)**[17]	273	100	98.66 to 100.00	0.043	23.5
**Krstic *et al*. (2017)** [18]	12689	0.449	0.34 to 0.58	1.97	25.48
**Total (fixed effects)**	642801	0.0696	0.063 to 0.076	100	100
**Total (random effects)**	642801	15.376	8.80 to 23.38	100	100

**Table 4B:** Test for heterogeneity

Q	2783.729
DF	3
Significance level	p<0.0001
I^2^ (inconsistency)	99.89%
95% CI for I^2^	99.87 to 99.91

### Prevalence of D variants

Table 5A shows 4 studies that evaluated the prevalence of the D variant included in the meta-analysis. The studies grouped different variants, including weak D-type 1, weak D-type 2, weak D-type 3, weak partial D, DAR, and other alleges. The narrow CI range indicates greater precision of the studies except for Krstic *et al*. [18], which showed a higher range of CI indicating lesser precision. Because of the larger study population, a higher percentage of fixed weight was assigned to Clarke *et al*. [16] in the fixed model. The studies showed a high degree of heterogeneity between weak D-type 1 variant (I^2^=92.70%; p<0.0001), weak D-type 2 variants (I^2^=91.86%; p<0.0001), weak D-type 3 (I^2^=93.06%; p<0.0001), weak partial D (I^2^=98.5%; p<0.0001), DAR (I2=97.53%; p<0.0001) and other variants (I^2^=98.66%; p<0.0001). The general prevalence of the D variant was 15% (95% CI, 9%–23%), and the prevalence of the D variant ranged from 0.05% to 100%, showing a high degree of heterogeneity between studies (I^2^=99.89%, p<0.0001) (Table 5B).

**Table 5A:** Prevalence of D variant

Study		Bub *et al*. (2016) [15]	Clarke *et al*. (2016) [16]	Laget *et al*. (2019) [17]	Krstic *et al*. (2017) [18]	Total (fixed effects)	Total (random effects)
Sample size		104	330	273	57	764	764
Proportion (%)	Weak D-type 1	8.654	36.061	31.136	42.105	30.442	28.378
	95% CI	4.034 to 15.793	30.874 to 41.500	25.691 to 36.995	29.143 to 55.916	27.203 to 33.832	16.687 to 41.777
Proportion (%)	Weak D-type 2	11.538	13.636	22.344	0	14.66	10.713
	95% CI	6.106 to 19.288	10.123 to 17.818	17.544 to 27.756	0.000 to 6.267	12.233 to 17.362	3.938 to 20.281
Proportion (%)	Weak D-type 3	1.923	14.848	5.861	31.579	10.255	11.375
	95% CI	0.234 to 6.774	11.191 to 19.151	3.387 to 9.343	19.905 to 45.243	8.200 to 12.620	3.927 to 22.029
Proportion (%)	Weak D partial	49.038	0	12.088	17.544	7.56	15.028
	95% CI	39.102 to 59.031	0.000 to 1.112	8.469 to 16.555	8.747 to 29.906	5.792 to 9.663	0.806 to 42.124
Proportion (%)	DAR	11.538	0	19.048	0	5.048	5.018
	95% CI	6.106 to 19.288	0.000 to 1.112	14.565 to 24.216	0.000 to 6.267	3.610 to 6.843	0.0210 to 20.217
Proportion (%)	Other weak D	7.692	35.455	9.524	92.982	25.093	34.625
	95% CI	3.379 to 14.595	30.293 to 40.880	6.316 to 13.643	82.996 to 98.055	22.062 to 28.317	8.924 to 66.623
Weight (%)	Fixed	13.67	43.1	35.68	7.55	100	100
	Random	24.93	25.3	25.27	24.5	100	100

**Table 5B:** Test for heterogeneity

	Weak D-type 1	Weak D-type 2	Weak D-type 3	Weak partial D	DAR	other variants
Q	41.0712	36.8425	43.2073	200.1092	121.319	223.6652
DF	3	3	3	3	3	3
Significance level	p<0.0001	p<0.0001	p<0.0001	p<0.0001	p<0.0001	p<0.0001
I^2^ (inconsistency)	92.70%	91.86%	93.06%	98.50%	97.53%	98.66%
95% CI for I^2^	84.52 to 96.55	82.34 to 96.25	85.44 to 96.69	97.62 to 99.06	95.74 to 98.56	97.90 to 99.14

### Distribution of RhD positive, discrepant results, and variant D among pregnant women

**Figure 2:**
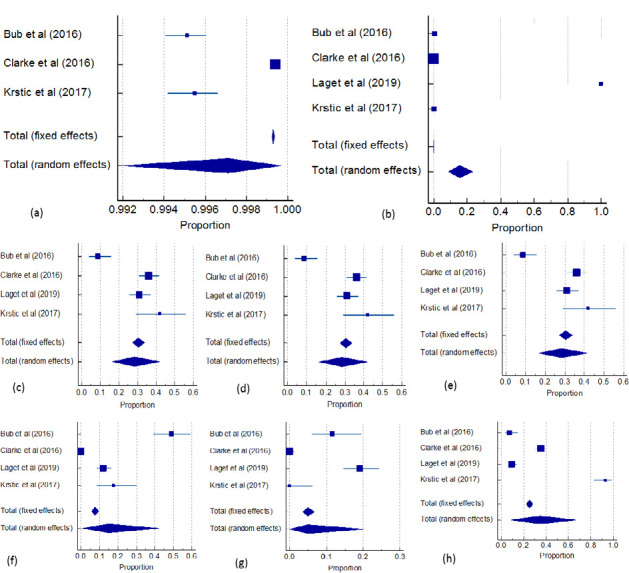
Forest plots for the distribution of RhD positive, discrepant results, and D variant among pregnant women. (a) variation in RhD positive prevalence, (b) serological discrepant results, (c)(d) and (e) weak D-type 1, type 2, and type 3 variants, (f) weak partial D type, (g) DAR variants, (h) other variants

The pooled distribution of RhD-positive, discrepant results, and the D variant is presented on the forest plots. These plots provide a visual representation of the selected studies included in the meta-analysis and the degree of variation among their results. The area of blue squares represents the weight of each study, and the horizontal line extends their 95% CI. The overall weighted average effect for the fixed and random models, along with their respective CIs, is represented by a diamond shape at the bottom of the plot. With respect to RhD +ve prevalence, the larger box size for Clarke *et al*. [16] indicates a larger population than in other studies. The random effect includes the line of no effect, and hence, no significant effect was noticed in the study. However, individual studies show significant variation with high heterogeneity (Figure 2a). In the case of serological discrepant results, Laget *et al*. [18] show significant variation with a higher proportion of variation compared to other studies. The pooled random and fixed effects show more significant heterogeneity in the current study (Figure 2b). Figure 2 (c-e) shows that weak D-type 1, type 2, and type 3 variants are common with no significant difference except for Bub *et al*. [15]. Even the fixed and random models specify that these variants are widely prevalent in the study. However, the weak partial D type significantly varied between studies, but the pooled effect is common (Figure 2f). The prevalence of DAR variants varied among individual studies showing a high level of heterogeneity (Figure 2g). Conversely, when considering other variants, the individual studies demonstrated high variation but with greater precision. However, the pooled random effect for these variants did not yield a significant result (Figure 2h). Overall, a high level of heterogeneity was observed for all the variants assessed in the study.

### Publication bias

**Figure 3:**
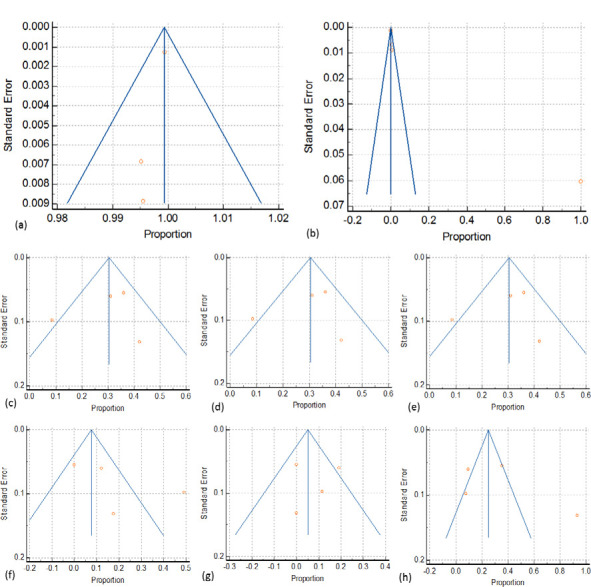
Funnel plot with 95% confidence limits for RhD-positive, discrepant results and D variant among pregnant women. (a) variation in RhD positive prevalence, (b)serological discrepant results, (c)(d) and (e) weak-D type2, type 2 and type 3 variants, (f) weak partial D type, (g) DAR variants, (h) other variants

Figure 3 (a-h) represents the funnel plots used to assess publication bias. A clear, apparently asymmetric funnel plot is noticed in all cases. Despite this asymmetry, the p-values from Egger's regression test are all >0.05 for all variants, indicating no evidence of publication bias (Table 6). The asymmetric funnel represents the true heterogeneity among the studies.

**Table 6:** Publication bias

Egger’s test	Intercept	95% CI	Significance p level
RhD-positive	-13.8181	-47.1867 to 19.5505	0.1196
Discrepant	29.6974	-26.8860 to 86.2807	0.1525
Weak D-type 1	-3.4917	-32.5895 to 25.6060	0.6571
Weak D- type 2	-7.4716	-26.0189 to 11.0756	0.2252
Weak D- type 3	1.6576	-29.7113 to 33.0265	0.8413
Weak partial D	15.4234	-34.3077 to 65.1544	0.3137
DAR	0.9501	-52.2099 to 54.1100	0.9457
Other variants	10.1731	-55.1526 to 75.4989	0.5718

## DISCUSSION

Anti-D alloimmunization in pregnant women can be prevented by anti-D prophylaxis. However, this depends on the precise establishment of the RhD status of pregnant women [19]. According to the British Committee for Standards in Hematology guidelines (BCSH), RhD-negative pregnant women not previously sensitized are eligible for anti-D prophylaxis either with a single dose at 28 weeks or two doses at 28 and 34 weeks [20]. Many laboratories have different methods for interpreting RhD results, depending on the serological antibodies used for the Figure 2: Forest plots for the distribution of RhD positive, discrepant results, and D variant among pregnant women. (a) variation in RhD positive prevalence, (b) serological discrepant results, (c)(d) and (e) weak D-type 1, type 2, and type 3 variants, (f) weak partial D type, (g) DAR variants, (h) other variants typed and grouped as RhD-positive without further molecular testing. Some of these variants have been linked to anti-D alloimmunization. Women with weak D mistakenly grouped as RhD positive do not qualify for anti-D prophylaxis, putting them at risk of anti-D alloimmunization and their fetus to HDNB. However, some variants can also be mistakenly grouped as RhD negative in the case of pregnancy; these women are, therefore, eligible for anti-D prophylaxis.

In certain countries, guidelines for preventing anti-D alloimmunization state that prophylaxis is unnecessary for women with weak D or Du phenotypes. At the same time, more recent recommendations emphasize the importance of clarifying ambiguous RhD typing results and treating all D variants other than weak D types 1, 2, and 3 as D negative [21]. Recent research in the USA has demonstrated that performing RHD genotyping for pregnant women with serologically weak D phenotypes is a cost-neutral strategy, with potential cost savings expected to increase over time [22]. It is necessary to highlight the relevance of serologic typing alongside the undeniable significance of RHD genotyping in resolving inconsistent RhD typing results and identifying D variants. If D variants are not recognized during the initial serologic typing, and carriers of D variants are mistakenly classified as RhD positive, the need for genotyping may go unnoticed. A study by Krstic *et al*. [1] reported that immunoprophylaxis was initiated for women identified as partial D carriers through genotyping when the serologic RhD typing results were unclear in 2008. To accurately identify partial D carriers that require immunoprophylaxis, other studies also highlight the significance of genotyping RHD in pregnant women with variant antigen D [7, 20]. We included a low number of studies for meta-analysis, which is the main limitation of the review. Despite this limitation, the current systematic review and meta-analysis provides an evidence-based report on anti-D alloimmunization among pregnant women with RhD variants and evaluates the clinical consequences of anti-D alloimmunization. The fact that genotyping was not done on alloimmunized RhD-negative women is another limitation of our study.

## CONCLUSION

Anti-D immunization could occur in pregnant women with RhD variants. All four studies focused on molecular testing of samples with discrepant or weak results with at least two anti-D antibodies using serological methods. These findings highlight the prevalence of serological discrepancies associated with different types of D variants, which can potentially alter the risk of hemolytic disease in the fetus and newborn. The results also reveal significant heterogeneity in RhD typing among the included studies, indicating challenges in determining the prevalence of D variants and their impact on hemolytic disease. The fixed- and random-effects models were applied to infer a high degree of variation among the studies. Although there is no evidence of publication bias, the high level of heterogeneity noticed encourages more studies to pay attention to D variants and their prevalence.

## Data Availability

Data is available from the corresponding author upon request.
